# Caregiver Characteristics of Adults with Acute Traumatic Brain Injury in the United States and Latin America

**DOI:** 10.3390/ijerph19095717

**Published:** 2022-05-07

**Authors:** Shannon B. Juengst, Paul B. Perrin, Daniel W. Klyce, Therese M. O’Neil-Pirozzi, Susan Herrera, Brittany Wright, Jean Lengenfelder, Kirk Lercher, Librada Callender, Juan Carlos Arango-Lasprilla

**Affiliations:** 1 Department of Physical Medicine & Rehabilitation, University of Texas Southwestern Medical Center, Dallas, TX 75390, USA; susan.herrera@utsouthwestern.edu (S.H.); brittany.wright@utsouthwestern.edu (B.W.); 2 TIRR Memorial Hermann Brain Injury Research Center, Houston, TX 77030, USA; 3 Department of Physical Medicine & Rehabilitation, UT Health Sciences Center at Houston, Houston, TX 77030, USA; 4 Department of Psychology, Virginia Commonwealth University, Richmond, VA 23284, USA; pperrin@vcu.edu; 5 Department of Physical Medicine and Rehabilitation, Virginia Commonwealth University, Richmond, VA 23284, USA; 6 Central Virginia Veterans Affairs Health Care System, Richmond, VA 23249, USA; daniel.klyce@va.gov; 7 Virginia Commonwealth University Health System, Richmond, VA 23284, USA; 8 Sheltering Arms Institute, Richmond, VA 23233, USA; 9 Department of Physical Medicine and Rehabilitation, Spaulding Rehabilitation Hospital, Boston, MA 02129, USA; t.oneil-pirozzi@neu.edu; 10 Department of Communication Sciences and Disorders, Northeastern University, Boston, MA 02115, USA; 11 Department of Physical Medicine & Rehabilitation, Rutgers-New Jersey Medical School, Newark, NJ 07101, USA; jlengenfelder@kesslerfoundation.org; 12 Kessler Foundation, East Hanover, NJ 07936, USA; 13 Department of Physical Medicine & Rehabilitation, JFK Johnson Rehabilitation Institute, Hackensack Meridian Health, Edison, NJ 08820, USA; kirk.lercher@gmail.com; 14 Baylor Scott & White Institute for Rehabilitation, Dallas, TX 75246, USA; librada.callender@bswhealth.org; 15 BioCruces Bizkaia Health Research Institute, 48903 Barakaldo, Spain; jcalasprilla@gmail.com; 16 IKERBASQUE, Basque Foundation for Science, 48009 Bilbao, Spain; 17 Department of Cell Biology and Histology, University of the Basque Country, 48940 Leioa, Spain

**Keywords:** traumatic brain injury, caregivers, Latin America, cross-cultural, acute

## Abstract

**Objectives**: To compare characteristics of caregivers of adults with acute traumatic brain injury (TBI) in the U.S. and Latin America (Mexico and Colombia). **Design**: Secondary data analysis of two cohorts. Cohort 1: English-speaking caregivers of adults with TBI in the U.S. (n = 80). Cohort 2: Spanish-speaking caregivers of adults with TBI in Mexico or Colombia (n = 109). **Results**: Similarities between the U.S. and Latin American caregiver groups, respectively, were: predominantly women (81.3%, 81.7%, respectively); spouses/domestic partners (45%, 31.2%); and motor vehicle accident (41.5%, 48.6%) followed by fall etiologies (40%, 21.1%). Differences between U.S. and Latin American caregivers were: age (49.5 years, 41.5 years, *p* < 0.001); employment status ((*Χ*_5_^2^ = 59.63, *p* < 0.001), full-time employment (63.7%, 25.7%), homemaker (2.5%, 31.2%), and retired (17.5%, 1.8%)); violence-related etiology (2.5%, 15.6%); and severity of depressive symptoms (*M* = 7.9, *SD* = 5.8; *M* = 5.8, *SD* = 5.7; *p* = 0.014). **Conclusions**: TBI caregivers in the U.S. were older and employed full-time or retired more often than those in Latin America. Violence-related etiology was nearly five times more common in Latin America, raising concerns for potential implications of post-traumatic stress and family adjustment after injury. Although both groups likely could use mental health support, this was particularly true of the U.S. cohort, maybe due to differential demographics, mechanisms of injury, or family and community support.

## 1. Introduction

Traumatic brain injury (TBI) is the leading cause of death and disability in young people worldwide [1]. In Latin America, there are 909 TBIs per 100,000 persons annually [2], whereas in the U.S. and Canada there are 1299 TBIs per 100,000 persons annually [3]. Reasons for higher mortality in low- and middle-income countries (LMICs) include lack of injury prevention regulations and higher frequency of risk factors associated with TBI (e.g., young age, poverty, and violence) [4,5,6]. Clinical care for TBI in LMICs presents unique challenges including long transit times from injury to hospital, a high proportion of motorcycle and pedestrian accidents, lack of standardized medical care, and limited rehabilitation services [3,6,7,8]. Further, access to clinical care can be further complicated in LMICs, such as many in Latin America, where 30.1% of individuals live below the poverty line [9] as compared to higher income countries, such as the U.S., where 11.4% of the population lives below the poverty line [10,11].

TBI occurs globally, affecting not only the individual who sustained the injury but also those close to them (e.g., family, partners, and friends). After moderate-to-severe injury, those in a person with TBI’s personal network often assume a role of caregiver or care partner, supporting and assisting the individuals with TBI with ongoing functional needs and activities that they themselves can no longer manage independently. Caregiver burden associated with this support is well recognized internationally, with some evidence that burden increases over time [12,13]. Many caregivers report being ill-prepared to take on a caregiver role [14,15]. Clinical care challenges translate into greater needs for care and support, which often fall to caregivers. Individuals who survive a TBI often experience cognitive (e.g., attention deficits, memory issues, language difficulties), emotional (e.g., emotional regulation difficulties, anxiety, depression), and social (e.g., inappropriate social and behavioral) symptoms, requiring ongoing support [16]. Approximately two thirds of needs and activities of individuals with TBI are provided by family or friends after hospital discharge [17]. Despite this, caregivers of adults with TBI, in both the U.S. and Latin America, often report unmet needs, including lack of resources, resulting in caregiver strain and poor health [18,19,20,21]. Caring for and supporting an individual with TBI can result in an overall reduced quality of life [22,23].

Caregivers also bring their own histories, rituals, and traditions to the caregiving role, and while caregivers provide care worldwide, cultural norms differentially impact characteristics of caregivers and their caregiving roles. For example, familism, the importance of placing family needs before individual interests, is valued significantly more in Latin America than in the U.S. [24]. In contrast to U.S. communities, some of which view caring for elders as an obligation and a role that family members struggle to assume, Latino communities often view caring for elders as an honor and a role that family members more willingly assume [25]. Despite these cultural differences, existing studies on caregiver needs and characteristics primarily focus on individuals in high-income regions (e.g., U.S), whereas caregivers in LMICs (i.e., Latin America) have not received similar attention [10].

While caregiver burden is a universally documented consequence of TBI, cross-cultural comparisons of TBI caregivers are infrequent. Insight into cultural caregiving universalities and differences would inform needed caregiver-centered education, counseling, training, and other supports. As a first step in providing this much-needed insight, our objective was to compare personal characteristics and care recipient factors between caregivers of adults admitted to inpatient rehabilitation for TBI in the United States (U.S.) and Latin America (Mexico and Colombia). To date, no research has yet to compare the characteristics of TBI caregivers across these global regions, nor to examine potential differences in their depressive symptoms. Potential differences in these variables by global region could highlight the unique characteristics and needs of TBI caregivers through a cross-cultural lens, which would better help tailor services for these medically underserved but critical group of individuals.

## 2. Methods

### 2.1. Design and Participants

This was a secondary data analysis of cohorts from two studies [7,26,27]. Cohort 1 included English-speaking caregivers of adults with acute TBI in the U.S. (n = 80) recruited between November 2018 and June 2021. Cohort 2 included Spanish-speaking caregivers of adults with acute TBI in Mexico or Colombia (n = 109) recruited between July 2014 and June 2017. Support services—for both individuals with TBI and their caregivers—are highly variable, both in the U.S. and Latin America. There is tremendous variability across countries, states, regions, and managed care approaches; highly detailed data on specific services and supports received by participants were unavailable. However, it could broadly be stated that individuals with TBI from the U.S. had the opportunity to receive somewhat more comprehensive inpatient rehabilitation services (e.g., occupational therapy, neuropsychology, etc.) than those from Latin America. In both global regions, most informal caregiving after discharge relies on unpaid work of family members, and there are few, if any, formal support services or governmental designations for caregivers. In both parent studies, all participants provided informed consent, and all study procedures were approved and supervised by each participating site’s Institutional Review Board.

**Cohort 1:** English-speaking caregivers of adults with acute TBI in the U.S. (TBIMS).

Caregivers of individuals with TBI in the U.S. were recruited as part of a large study examining the feasibility of a problem-solving intervention from three Traumatic Brain Injury Model Systems (TBIMS) [28] Centers (in Texas and New Jersey) prior to their care recipient’s discharge from inpatient rehabilitation. Caregivers had to (a) be over 18 years old, (b) have longer than a one-year relationship with the care recipient, (c) be able to communicate fluently in English, (d) be cognitively able to make decisions and provide informed consent, and (e) not be in a legal dispute with family over the individual’s capacity to be a caregiver. Caregivers completed the survey, as part of the baseline assessment of the parent study, in person at the rehabilitation centers or remotely via telephone or electronic questionnaire.

**Cohort 2**: Spanish-speaking caregivers of adults with acute TBI in Mexico or Colombia (n = 109).

TBI caregivers from Latin America were recruited as part of a larger study examining TBI caregiver needs over the acute hospitalization time period and several months after discharge. Caregivers were recruited from three hospitals in Mexico City, Mexico (n = 68) and Cali (n = 21) and Neiva (n = 20), Colombia. Caregivers had to (a) be related to a person with TBI aged 18 or older via blood or marriage and/or be a close friend, (b) live in the household with the person with TBI, (c) be able to communicate orally in Spanish, and (d) be the primary caregiver providing active daily care for the person with TBI. They were excluded if they had a history of neurological conditions, serious psychiatric disorders, or current and serious alcohol or drug abuse. All caregivers completed the survey orally while the person with TBI was still in the hospital.

### 2.2. Measures

Upon their enrollment in both studies, caregivers provided their age, gender, relationship with the care recipient, marital status, employment status, and their care recipient’s TBI etiology. Participants also completed the Patient Health Questionnaire 9 (PHQ-9). The PHQ is a 9-item measure of depressive symptoms experienced in the past two weeks, validated in both English and Spanish [29,30]. Caregivers endorsed items on a 0–3 scale with 0 indicating that they experienced symptoms “not at all” in the past two weeks and 3 indicating they have experienced them “nearly every day.” Total scores for the PHQ range from 0–27, with scores of 0–4 indicating no depression, 5–9 indicating mild depression, 10–14 indicating moderate depression, 15–19 indicating moderately severe depression, and 20+ indicating severe depression [29].

### 2.3. Data Analysis

We calculated summary statistics to characterize caregivers in each cohort, including means and standard deviations for age and depressive symptoms and numbers and percentages for all other variables. We compared age and depressive symptoms between cohorts using unpaired t-tests. Categorical variables were compared using chi-square tests.

## 3. Results

See Table 1 for all summary and group comparison statistics. U.S. caregivers were significantly older than Latin American caregivers (*d* = 0.57). Employment status differed significantly, with most U.S. caregivers being employed full-time followed by retired, whereas the most common employment status for Latin American caregivers was homemaker followed by full-time and part-time employment. TBI etiologies in the two cohorts different significantly. The most common etiologies in both cohorts were motor vehicle accidents and falls, though falls occurred more frequently in the U.S. than Latin America. Violence-related etiologies were much more common in Latin America compared to the U.S. Caregivers in both samples endorsed mild depressive symptoms, but those in the U.S. endorsed significantly greater depressive symptom severity (*d* = 0.37).

## 4. Discussion

This study compared characteristics of caregivers of adults with acute TBI in the U.S. and Latin America. Both caregiving groups tended to be predominantly women and spouses/domestic partners of the person with TBI. The mechanisms of injury in both groups tended to be motor vehicle accidents followed by falls, though violence was a more common etiology in Latin America. Caregivers in the U.S. tended to be older than those in Latin America and more often had full-time employment or were retired, whereas those in Latin America more so tended to be homemakers and also showed lower levels of depression symptoms.

The finding that caregivers were equally highly likely to be women in both samples generally suggests that traditional gender roles surrounding caregiving may be operating in both global regions with women more often assuming caregiving roles than men. This is consistent with previous research finding that across a wide range of disabilities and chronic health conditions, women are the family members who tend to take on caregiving roles [31,32]. Although this has been shown to be particularly true in Latin America given rigid gender roles and other traditional duties for women within families [33], the current study did not find that women were more likely to be caregivers in Latin America vs. the U.S. This could in part be due to the region of the U.S. where the data were collected (Texas) which also may have strong traditional gender roles. The finding that there were no global differences in marital status of the caregiver may similarly reflect comparable gender roles across the specific regions.

For both people with TBI and their caregivers, the experience of brain injury confers elevated risk for negative mental health outcomes across many populations and cultural contexts [18,34,35,36,37,38,39,40]. Among the mental health concerns that can develop, PTSD is commonly comorbid with TBI [41]. The Latin American community may be particularly vulnerable to trauma-based outcomes, given the five-times higher rates of violence as a mechanism of injury relative to the U.S, even as the posttraumatic effects of injury due to violence reverberate throughout other members of the family. There is evidence to suggest that participation in inpatient rehabilitation for TBI can mitigate negative posttraumatic outcomes, though these services are not consistently available in Latin American health care systems and rarely extend to family caregivers or others who might experience vicarious trauma [19]. Although the effects of violence-related TBI on caregivers are studied predominantly in the context of Veteran samples or military-service-related injuries [42,43], these studies suggest that caregivers are at risk for negative outcomes associated with burden and emotional functioning—and possibly secondary trauma [44,45]. Although we assessed symptoms of depression among caregivers, we did not assess symptoms of posttraumatic stress, which is especially important both among people with a history of TBI related to violence and their caregivers.

Conversely, although caregivers in both the U.S. and Latin America could benefit from mental health support for depression, this may be particularly true for U.S. caregivers. There are several potential reasons that U.S. caregivers in our study may have reported more severe depressive symptoms, including (but not limited to) factors related to symptom-reporting (e.g., stigma of depression, not wanting to be seen as “complaining”) or to cultural roles. Often learned from the family, Latinos tend to suffer without complaint (stoicism) to maintain levels of functioning. Individuals who “complain” about fulfilling social duties sometimes are regarded as weak, unable to bear suffering with courage and pride [46]. Additionally, the expectation to sacrifice oneself for the care of loved ones (marianismo) can further impact reports of depression due to the social stigma surrounding mental health issues and help-seeking behaviors [47]. For example, familism—the importance of placing family needs before individual interests—is valued to a greater degree in Latin America than in the U.S. [24]. In contrast to U.S. communities, some of which view caring for elders as an obligation and a role that family members struggle to assume, Latino communities sometimes view caring for elders as an honor and a role that family members willingly assume [25]. The heavy influence of familism has led to many Latin American caregivers, whether they currently reside in Latin America or the U.S., not even to self-identify as caregivers [48]. In Latin America, considering cultural implications that women are expected to assist the family, female caregivers may also feel more prepared for caregiving roles than men [22]. Men have reported higher caregiver burden than women, suggesting the need for caregiver interventions that are gender-informed [22]. Therefore, ongoing work is needed to identify how culture-based gender roles interact with other cultural values, such as familism, to affect caregivers’ mental health.

Caregivers in the U.S. were more frequently employed full-time in the current study, whereas Latin American caregivers were more often homemakers, unemployed, or employed part-time. This may result in more difficulty balancing life roles for U.S. caregivers. Balancing work and caregiving can be highly demanding and requires individuals to divert time, energy, and financial resources to caregiving [49]. Balancing life roles between family and work is well-discussed in the literature, as working individuals are increasingly caring for children, elders, and family members with disabilities [50]. Additionally, caregivers in the U.S. tended to be older in comparison to those from Latin America. Older caregivers are reported to have poorer health and more physical distress and to engage less often in self-care compared to younger caregivers [51,52].

Falls account for an increasing number of TBIs as the population ages [53] (70% of all TBIs in people over 65 [53]); almost half of all TBI-related hospitalizations in the U.S. are now related to falls, surpassing motor vehicle accidents [54]. Epidemiological studies in other parts of the world reflect a similar shift in the etiology of TBI toward falls in concert with an increasingly aging population [55,56], though motor vehicle accidents continue to be the most common mechanism of TBI in LMICs [57]. This is consistent with our findings that, while falls were the second most common cause of injury in both cohorts, they were far more common in the U.S. than in Latin America. This may have a significant impact on healthcare utilization as older adults are also more likely to accumulate more comorbid conditions that can have an adverse effect on their recovery from TBI and result in more prolonged hospitalizations [53]. The implications from older patients with increasing medical complexity being admitted to inpatient rehabilitation facilities and the impact this may have on other patients remain to be seen. Additionally, access to acute inpatient rehabilitation facilities varies in different parts of the world, and an increasing global aging population may prove challenging for regional health care systems [58].

Violence is a leading cause of injury among people with TBI around the world [10] and a leading cause of death in many Latin American countries, including Colombia and Mexico [13,59]. Violence was an etiological factor that affected five times as many people with TBI in the Latin American sample compared to the U.S. sample. A recent systematic scoping review of TBI as a result of violence concluded there are consistent pre-injury risk factors associated with violent injury [60], including male gender, problematic substance use, interactions with the legal system, unemployment, and—only in the U.S.—being non-White; although long-term outcomes were comparable to other mechanisms of injury, people with violence-related TBI tended to have lower levels of functional independence during the inpatient rehabilitation phase of recovery, as well as lower levels of community participation productivity one year post-injury [60]. A previous study using the TBIMS national database found that violence-related injuries tend to be associated with higher levels of disability and caregiver burden [61]. However, aside from this study and studies related to marital stability following TBI [62,63], the Bates et al. review (2016) [60] concluded that there was a notable lack of research regarding the effects of violence-related injuries on informal caregivers.

### Limitations and Future Directions

The results of this study should be interpreted in the context of the following limitations. First, the Latin America sample was from two Latin American countries (Mexico and Colombia), and as a result the findings cannot necessarily be generalized to caregivers of other Latin American countries. Additionally, the U.S. sample included caregivers who identified as Hispanic, which makes speculation about differences related to cultural values less clear. We cannot broadly generalize cultural values to all individuals from a particular ethnic group, as “culture” does not equate with racial or ethnic group. Individuals within the broader Hispanic/Latino population—both in Latin American and in the U.S.—represent a diversity of cultures and health-related needs [64]. Additionally, Hispanic/Latino caregivers who move to or who were born in the U.S. are influenced to assimilate into the U.S. culture, especially the younger ones [48]—approximately 27% of family caregivers in the U.S. identify as Hispanic/Latinx and millennial [65]. Cultural differences between U.S. and foreign-born Latino caregivers can shape the exchanges between care recipients and caregivers uniquely [65]. Second, it is common in Latin American countries that younger people assume caregiving roles; therefore, the results may not generalize to caregivers younger than 18 years of age. Third, some of the differences found in this study between the two groups could be explained by variables not available for analyses, such as time as a caregiver, TBI severity, patient access to rehabilitation services, social support, family income, reimbursement for caregiving or other caregiver supports, and access to community resources and other support. Fourth, in this study, we only explored select sociodemographic and injury characteristics and depression symptoms. Future studies should explore other similarities and differences in mental health outcomes (e.g., burden, anxiety, stress, etc.) and psychosocial characteristics (e.g., quality of life, satisfaction with life, social support, etc.). Fifth, this was a cross-sectional study, and it is unknown whether the presence of mild symptoms of depression is the same in the two samples over time. Future longitudinal studies should be conducted looking at similarities and differences in mental health outcomes over time between these two groups. Lastly, inclusion criteria for the two cohorts were not identical, with the two most notable differences being that in the Latin American cohort, caregivers had to be residing with the care recipient and be the primary person responsible for assisting the care recipient with daily activities, whereas those in the U.S. cohort did not have to reside with the person with TBI or had to be responsible in any way for the health or well-being of the person with TBI. The relationship between residence and depression status is complex and has been found to differ as a function of White versus Black race [66], so further study is necessary to examine how residing with a care recipient affects caregivers across different cultural backgrounds. Because participant enrollment rate was not systematically tracked across all participants, it is unknown how these variables play into which caregivers decided to enroll in the study.

## 5. Conclusions

While need for caregiver support post-TBI is universal, caregiver characteristics are not. In Latin America, caregivers post-TBI were more varied in their primary life roles, whereas U.S. caregivers more often juggled caregiving responsibilities with full-time employment. Caregivers in the U.S. were older and employed full-time or retired more often than those in Latin America. Their care recipients were also far more likely to be injured in a fall, which may reflect an older age among individuals with TBI as well. Violence-related etiology (excepting gunshot wound) was nearly five times more common in Latin America than the U.S., raising concerns for potential implications of posttraumatic stress recovery and family adjustment after injury [67,68]. Though both groups endorsed mild depressive symptoms and could likely benefit from mental health support, caregivers in the U.S. reported more symptoms than those in Latin America. This is consistent with past research suggesting that the impact of caregiver strain on mood may differ across cultures in part due to differential demographics and mechanisms of injury. These findings generally suggest that caregivers from the U.S. may have particularly accentuated levels of depression symptoms and therefore are especially likely to benefit from evidence-based, caregiver-tailored depression treatment. Conversely, because individuals with TBI in Latin America were more likely to sustain their injury via violence, posttraumatic stress assessment and treatment may be needed for caregivers in this global region. Because caregivers in the U.S. were more likely to be employed, interventions geared toward helping caregivers manage work life and caregiving life may be helpful. Future research is needed on the development and implementation of these types of caregiver interventions in order to improve outcomes for both TBI caregivers and their care recipients.

## Figures and Tables

**Table 1:** Participant Characteristics.

Characteristic	U.S. Caregivers (English) n = 80	Latin American Caregiver (Spanish) n = 109	Statistical Comparison
Age, mean (SD)	49.5 (14.0)	41.5 (13.9)	t_187_ = 3.90, *p* < 0.001 *
Gender (English)/Sex (Spanish), *n* (%)			*Χ*_1_^2^ = 0.0049, *p* = 0.94
Men/Male	15 (18.8)	20 (18.3)	
Women/Female	65 (81.3)	89 (81.7)	
Role-type of Caregiver, *n* (%)			*Χ*_4_^2^ = 10.48, *p* = 0.03 *
Spouse/partner	36 (45.0)	34 (31.2)	
Parent	18 (22.5)	29 (26.6)	
Child	19 (23.8)	21 (19.3)	
Sibling	2 (2.5)	14 (12.8)	
Other relationship	4 (5.1)	11 (10.1)	
Injury Mechanism for TBI Survivor, *n* (%)			*Χ*_5_^2^ = 14.98, *p* = 0.01 *
Motor vehicle accident	33 (41.5)	53 (48.6)	
Fall	32 (40.0)	23 (21.1)	
Pedestrian or bicycle accident	6 (7.5)	10 (9.2)	
Violence	2 (2.5)	17 (15.6)	
Gunshot wound	2 (2.5)	2 (1.8)	
Other/Unknown	5 (6.3)	4 (3.7)	
Marital status, *n* (%)			*Χ*_3_^2^ = 6.96,
Single	12 (15.0)	19 (17.4)	*p* = 0.07
Married/Domestic Partner	56 (70.0)	60 (55.1)	
Divorced/Separated	11 (13.8)	21 (19.3)	
Other	1 (1.3)	9 (7.5)	
Current employment status, *n* (%)			*Χ*_5_^2^ = 59.63, *p* < 0.001 *
Full-time	51 (63.7)	28 (25.7)	
Part-time	8 (10.0)	22 (20.2)	
Homemaker	2 (2.5)	34 (31.2)	
Unemployed	3 (3.8)	18 (16.5)	
Student	2 (2.5)	5 (4.6)	
Pension/retired	14 (17.5)	2 (1.8)	
Depression (PHQ)	7.9 (5.8)	5.78 (5.74)	t_186_ = 2.48, *p* = 0.014 *

Note. * = *p* < 0.05.

## Data Availability

Data are available from the corresponding author upon request.
